# Draft Genome Sequence of *Streptomyces* sp. Strain PTY087I2, Isolated from *Styela canopus*, a Panamanian Tunicate

**DOI:** 10.1128/genomeA.00856-16

**Published:** 2016-09-15

**Authors:** Samantha M. Gromek, Anne A. Sung, Jonathan L. Klassen, Marcy J. Balunas

**Affiliations:** a Division of Medicinal Chemistry, Department of Pharmaceutical Sciences, University of Connecticut, Storrs, Connecticut, USA; b Department of Molecular and Cell Biology, University of Connecticut, Storrs, Connecticut, USA

## Abstract

*Streptomyces* sp. PTY087I2 is a marine bacterium isolated from *Styela canopus*, a tunicate collected in Bocas del Toro, Panama. Here, we report a draft genome sequence for this bacterium, found to have 94.7% average nucleotide identity (ANI) with *Streptomyces roseosporus* NRRL 11379, and containing a diverse suite of secondary metabolite gene clusters.

## GENOME ANNOUNCEMENT

Ascidians, commonly referred to as tunicates, are sessile organisms that harbor diverse microbial communities (1). These tunicate-associated bacteria have been shown to produce secondary metabolites to protect against harmful microorganisms and predators (2). *Streptomyces* spp. are frequently associated with tunicates and known to be a robust source of secondary metabolites with intricate structural moieties that often possess biological activity (3). We isolated *Streptomyces* sp. strain PTY087I2 from homogenized tissue of *Styela canopus*, a solitary tunicate collected in the Republic of Panama. Whole-genome sequencing revealed extensive secondary metabolite biosynthetic potential, prompting us to investigate the genome for biosynthetic gene clusters (BGCs) previously associated with known biological activity.

*Styela canopus* was collected from mangrove roots in Bastimentos National Park in Bocas del Toro, Panama (9°17.398′N 82°11.106′W), surface sterilized, homogenized, and plated onto R2A agar supplemented with Instant Ocean, cycloheximide, and nalidixic acid. *Streptomyces* sp. PTY087I2 was grown in liquid culture until turbid using yeast starch peptone medium supplemented with Instant Ocean. Genomic DNA was extracted from 1 ml of confluent culture using the Promega Wizard genomic DNA purification kit, according to the manufacturer’s protocol. Extracted DNA was quantified by measuring absorbance at 260 nm using a Thermo Scientific NanoDrop 2000c spectrophotometer. Subsequently, 200 ng of genomic DNA was fragmented, and adapter sequences were attached and size selected using an Illumina TruSeq library preparation kit, according to the manufacturer’s protocol. Our libraries were authenticated and the mean insert length was calculated using an Agilent Bioanalyzer high-sensitivity chip. The libraries were sequenced to 90× coverage on the Illumina MiSeq using the version 2 2 × 250-bp kit. The genome was assembled using A5-MiSeq pipeline version 20140113 (4), checked for contamination using the Blobology pipeline (5), and annotated using Prokka version 1.10 (6). ORFcor version 1.02 (7) and FastTree version 2.1.7 (8) were used to compare the genome to all other *Streptomyces* genomes in NCBI (as of 22 February 2016). The closest genome was *Streptomyces roseosporus* NRRL 11379, which had 94.7% average nucleotide identity (ANI) compared to *Streptomyces* sp. PTY087I2 (9, 10).

The *Streptomyces* sp. PTY087I2 genome contains 8,169,744 nucleotides in 84 scaffolds, with 71.5% G+C content and an *N*_50_ of 252,304 bp. The sequence was examined using Antibiotics and Secondary Metabolite Analysis SHell (antiSMASH) 3.0.5 to identify secondary metabolite gene clusters (11). There were 37 BGCs, including eight nonribosomal peptide synthetases (NRPS), four terpenes, three lantipepetides, two each of bacteriocin, ectoine, NRPS-type 1 polyketide synthase (T1PKS), siderophore, lassopeptide, T3PKS, and one each of melanin, T2PKS, butyrolactone, thiopeptide-lantipeptide, lantipeptide-melanin, T1PKS-NRPS, ladderane-arylpolyene, other KS-NRPS, other KS-T1PKS, and others. Of these clusters, seven shared 100% similarity with previously reported BGCs.

Interestingly, we identified one BGC with 83% homology to the granaticin cluster, and we confirmed the production of granaticin and several derivatives by this strain via liquid chromatography-mass spectrometry (LC-MS) (A.A. Sung, S. M. Gromek. M. J. Balunas, unpublished data). In the future, we plan to continue an investigation of this bacterium for the production of additional biologically active secondary metabolites.

### Accession number(s).

This whole-genome shotgun project has been deposited in DDBJ/ENA/GenBank under the accession no. LZRD00000000. The version described in this paper is the first version, LZRD01000000.
